# Evaluation of the Phase-Dependent Rhythm Control of Human Walking Using Phase Response Curves

**DOI:** 10.1371/journal.pcbi.1004950

**Published:** 2016-05-20

**Authors:** Tetsuro Funato, Yuki Yamamoto, Shinya Aoi, Takashi Imai, Toshio Aoyagi, Nozomi Tomita, Kazuo Tsuchiya

**Affiliations:** 1 Department of Mechanical Engineering and Intelligent Systems, The University of Electro-communications, Chofu, Tokyo, Japan; 2 Japan Science and Technology Agency (JST), CREST, Chiyoda-ku, Tokyo, Japan; 3 Department of Energy and Mechanical Engineering, Doshisha University, Kyotanabe-shi, Kyoto, Japan; 4 Department of Aeronautics and Astronautics, Kyoto University, Nishikyo-ku, Kyoto, Japan; 5 Department of Applied Analysis and Complex Dynamical Systems, Kyoto University, Sakyo-ku, Kyoto, Japan; Northeastern University, UNITED STATES

## Abstract

Humans and animals control their walking rhythms to maintain motion in a variable environment. The neural mechanism for controlling rhythm has been investigated in many studies using mechanical and electrical stimulation. However, quantitative evaluation of rhythm variation in response to perturbation at various timings has rarely been investigated. Such a characteristic of rhythm is described by the phase response curve (PRC). Dynamical simulations of human skeletal models with changing walking rhythms (phase reset) described a relation between the effective phase reset on stability and PRC, and phase reset around touch-down was shown to improve stability. A PRC of human walking was estimated by pulling the swing leg, but such perturbations hardly influenced the stance leg, so the relation between the PRC and walking events was difficult to discuss. This research thus examines human response to variations in floor velocity. Such perturbation yields another problem, in that the swing leg is indirectly (and weakly) perturbed, so the precision of PRC decreases. To solve this problem, this research adopts the weighted spike-triggered average (WSTA) method. In the WSTA method, a sequential pulsed perturbation is used for stimulation. This is in contrast with the conventional impulse method, which applies an intermittent impulsive perturbation. The WSTA method can be used to analyze responses to a large number of perturbations for each sequence. In the experiment, perturbations are applied to walking subjects by rapidly accelerating and decelerating a treadmill belt, and measured data are analyzed by the WSTA and impulse methods. The PRC obtained by the WSTA method had clear and stable waveforms with a higher temporal resolution than those obtained by the impulse method. By investigation of the rhythm transition for each phase of walking using the obtained PRC, a rhythm change that extends the touch-down and mid-single support phases is found to occur.

## Author Summary

Humans and animals tune their walking rhythms when motion is disturbed, such that they hesitate before making the transition from stance to swing phase. The effectiveness of rhythm control for stability has also been shown, and thus the elucidation of rhythm responses is important to understanding human strategies for walking control. In this research, how and when humans change their walking rhythm in response to disturbance is analyzed over the complete walking cycle. Phase response of human walking has previously been estimated by pulling the swing leg. The problem with this perturbation is that it hardly disturbs the stance leg, so here we apply the perturbation by changing floor velocity. However, perturbation from the floor yields another problem in that it weakly influences the swing leg, decreasing the precision of the PRC. The present research tackles this problem by introducing a new method for identifying rhythm characteristics by use of high-frequency perturbation, which allows us to obtain results with clear temporal resolution. We found that the human walking rhythm changes by lengthening the touch-down and mid-single support phases. These phase responses are compared with neural mechanisms for rhythm control, and relevance to the cutaneous and proprioceptive originated responses is shown.

## Introduction

Humans and animals control their walking rhythms to maintain their motion in a variable environment. When swing motion is disturbed during early swing phase, flexor muscles are evoked and swing phase is prolonged, and disturbance late in swing phase evokes extensor muscles, advancing touch-down (the stumbling corrective reaction [1–4]). Moreover, stimulation provided around the transition of stance to swing phase results in delayed initiation of swing phase [5, 6]. For these responses, cutaneous [7, 8] and proprioceptive [5, 9] afferents are engaged (for a review, see [10]), and the rhythm of CPG is reported to be shifted [11, 12]. Here, the actual response to the perturbation is determined not only by single afferents, but also by mutual influence of several sensory systems. For example, the swing leg receives presynaptic inhibition in proportion to loading on the contralateral leg [13, 14] and the proprioceptive response of the swing leg becomes one half that of the stance leg [15, 16]. Further global postural conditioning, such as the relation between the center of mass (COM) and COM velocity, is also reported to be related to reactions to the perturbation [17, 18]. Therefore, to elucidate the human response to disturbance, quantitative evaluation of the response to mechanical disturbances during walking is important.

For investigating the response to mechanical perturbation, walking experiments with various perturbations, such as sudden changes of treadmill belt speed [18], moving the floor forward and backward [17, 19–21] or right and left [22], or pulling legs [23, 24] or hips [25] have been performed. However, as pointed out by Feldman et al. [24], there are not many studies of phase responses to disturbances with various timings over the entire walking cycle. Feldman et al. [24] showed that walking rhythm is changed around the lift-off and mid-swing phases by pulling the ankle at many timings over the cycle of a few steps of walking, and Kobayashi et al. [26] and Nessler et al. [27] pulled legs during continuous walking to estimate the phase response curve, as explained below.

By focusing on the characteristics of walking as a stable rhythm, that is, as a limit cycle motion, walking motion can be approximately described in terms of a rhythm and its change (phase reduction). In the phase-reduction model, the response of the rhythm to perturbations applied at various timings in the cycle (phase, [28]) is described by a function of phase. This function, which depends on the perturbation timing, is called the phase response curve (PRC, also called the phase resetting curve; for a review, see [29]). PRC has been estimated for various biological rhythms, such as circadian rhythms [30, 31] and cardiac rhythms [32]. Perkel et al. [33] examined the rhythm response to single postsynaptic potential on pacemaker neurons of Aplysia and crayfish, and Pinsker [34] estimated the PRC of bursting neurons in an abdominal ganglion to investigate the modulation mechanism of bursting rhythms. As a PRC of locomotion rhythms, variation of relative phases among limbs in response to sensory input was investigated in stick insects [35] and cockroaches [36], and contributions of sensory input for coordinated stepping were shown. The PRC of walking cockroaches against physical disturbances was used with dynamical models for estimating the strength of inter-limb coordination [37]. In mammals, PRC of muscle activity during walking has also been investigated by using electrical stimulation of limb nerves [38].

From the estimated PRC of human walking [26], the contribution of rhythm control on the stability has been investigated. A dynamical simulation of a human skeletal model performed with changing rhythms (phase reset) in various timing showed stability enhancement by the phase reset [39]. Effective timing of the phase reset and the PRC of humans has also been indicated [40]. Phase resetting in response to the touch-down and lift-off timing was indicated from the behavior to proprioceptive afferents [41], and its functionality on the stability has been indicated by dynamical simulation of quadrupedal [42–45] and bipedal [45, 46] muscular-skeletal models and by experiments of biped [47–52] and quadruped [53] robots. To consider the control mechanism of walking, PRC provides information about when and to what extent rhythm should be controlled. PRC obtained by pulling swing legs [26, 27] are potentially insufficient to consider the relation between the transition of touch-down/lift-off timing and the PRC, because it is difficult for this stimulation to provide sufficient perturbation for the stance leg, and thus PRC around the swing/stance phase transitions are difficult to obtain. In the present research, floor velocity is changed as a perturbation, and the whole body, including the stance and swing legs, is moved by the perturbation.

Applying perturbation from the floor also generates another problem, in that the magnitude of the perturbation affecting the swing leg is small and response to the perturbation is possibly too small to obtain the PRC. To solve this problem, the present research introduces a new estimation method for PRC. When estimating the PRC from time-series data, the conventional method is the “impulse method,” which applies impulsive perturbations in various phases and investigates the magnitude of rhythm variation for each timing of single impulses (for example, [54, 55]). To estimate the PRC with high precision from measured time series, a method that uses sequential pulse perturbation instead of single impulse perturbation, the WSTA method, was recently proposed [56]. By applying this method to estimate the PRC of human walking, PRC for both the stance leg and the swing leg are expected to be obtained from the perturbation floor. Therefore, this research estimates the PRC of human walking using the WSTA method and then discusses the human rhythm control strategy deduced from the estimated PRC.

## Methods

### A. Ethics statement

This study was approved by the Ethics Committee of Doshisha University (1057). Written informed consent was obtained from all participants after the procedures had been fully explained.

### B. Estimation of PRC

To identify the PRC of human locomotion, we employ the WSTA method, which uses sequential impulse disturbance, instead of the method that evaluates rhythm variation against each disturbance applied as an intermittent impulsive perturbation. This subsection, based on Ota *et al*. [56], explains the PRC estimation procedure by using the WSTA method and constructs an experimental method for measuring the PRC of human walking.

#### Phase response curve (PRC)

In steady-state locomotion with cycle *T* and frequency *ω* ($\omega =\frac{2\pi }{T}$),if we assume the motion as a limit-cycle oscillator, the rhythm in the limit cycle is characterized by a phase *ϕ* such that $\frac{d\phi }{dt}=\omega$. Under a perturbation *I*(*t*), the phase *ϕ* changes according to the equation

$$\frac{d\phi }{dt}=\omega +Z\left(\phi \right)I\left(t\right),$$ (1)

where Z(*ϕ*) is the phase response curve (PRC) that represents the variation of rhythm against perturbation in each phase. The PRC expresses how the rhythm changes in response to perturbation, and so the human strategy for controlling rhythm is also considered to be reflected by the PRC. The physical units of phase *ϕ* and perturbation *I*(*t*) are radians and radians per second, respectively. *Z*(*ϕ*) is a dimensionless coefficient.

Now we consider the situation in which the perturbation *I*_*i*_(*t*) changes the walking cycle from *T* to *τ*_*i*_. Then, because the phase variation within one cycle (duration: *τ*_*i*_) is 2*π* ($\int_{0}^{\tau_{i}}d\phi =2\pi$), the integration of Eq (1) over *τ*_*i*_ leads to the relation

$$\frac{1}{2\pi }\int_{0}^{\tau_{i}}Z\left(\phi \right)I_{i}\left(t\right)dt=\frac{T-\tau_{i}}{T}.$$ (2)

The amount of phase shift by perturbation *I*_*i*_(*t*) for duration *T* can be calculated with the variable transformation $\tilde{t}=\frac{T}{\tau_{i}}t$.

$$\frac{1}{2\pi }\int_{0}^{T}Z\left(\phi \left(\tilde{t}\right)\right)I_{i}\left(\tilde{t}\right)d\tilde{t}=\frac{T-\tau_{i}}{\tau_{i}}=\Delta_{i}$$ (3)

In a human experiment, Δ_*i*_ of Eq (3) can be obtained by measuring the walking cycle against perturbation. Because the input perturbation *I*_*i*_ can be controlled, this enables the estimation of *Z*(*ϕ*) from the measured Δ_*i*_.

#### Estimation of PRC using the impulse method

To acquire a PRC from experimental data, impulsive stimulation has often been used. If the perturbation *I*_*i*_(*t*) is the Dirac delta function with positive value at time *s* and with magnitude *μ*_*I*_ (*I*_*i*_(*t*) = *μ*_*I*_*δ*(*t* − *s*)), Eq (3) becomes

$$\Delta_{i}=\frac{\mu_{I}}{2\pi }\int_{0}^{T}Z\left(\phi \left(\tilde{t}\right)\right)\delta \left(\tilde{t}-s\right)d\tilde{t}=\frac{\mu_{I}}{2\pi }Z\left(\phi \left(\text{s}\right)\right),$$ (4)

where *ϕ*(s) represents the phase corresponding to time *s*. Thus the phase response at phase *ϕ*(s) is

$$Z\left(\phi \left(\text{s}\right)\right)=\frac{2\pi }{\mu_{I}}\Delta_{i}.$$ (5)

Then, by applying such an impulse input for various phases *ϕ*(s) over the cycle, a phase response is obtained for the whole range of the walking cycle. However, this method requires one walking trial for each phase, and every trial requires an initial condition of stable locomotion to be established, which takes a long time. Thus the method takes a vast amount of time to obtain the PRC for the whole cycle. Because of fatigue in the human subject and the consequent variation of motion with time, the time for experimentation is limited, and so obtaining an accurate PRC of human locomotion via floor perturbation by this method is difficult.

#### Estimation of PRC using the WSTA method

The WSTA method has been proposed as an alternative to single impulse perturbation [56]. The pulsed perturbation used in WSTA, *I*_*i*_(*t*) = *μ*_*W*_*ξ*_*i*_(*t*) with magnitude *μ*_*W*_ and normalized sequence *ξ*_*i*_(*t*), is supposed to have no temporal correlation so that its auto-correlation is given by the Dirac delta function and the average perturbation among trials is 0. For such an input, the auto-correlation $\text{C}\left(\tilde{t},t\right)=\langle \left(I_{i}\left(\tilde{t}\right)-\langle I_{i}\left(\tilde{t}\right)\rangle \right)\left(I_{i}\left(t\right)-\langle I_{i}\left(t\right)\rangle \right)\rangle$ becomes

$$\text{C}\left(\tilde{t},t\right)=\mu_{W}{}^{2}\langle \xi_{i}\left(\tilde{t}\right)\;\xi_{i}\left(t\right)\rangle =\mu_{W}{}^{2}\delta \left(\tilde{t}-t\right),$$ (6)

where 〈·〉 represents an average over the trials. By multiplying *I*_*i*_(*t*) = *μ*_*W*_*ξ*_*i*_(*t*) into Eq (3) and by averaging it over trials, the following equation can be obtained.

$$\begin{array}{ll} \langle \Delta_{i}I_{i}(t)\rangle & =\frac{\mu_{W}{}^{2}}{2\pi }\int_{0}^{T}\langle Z\left(\phi \left(\tilde{t}\right)\right)\xi_{i}\left(\tilde{t}\right)\;\xi_{i}\left(t\right)\rangle d\tilde{t}\\ & =\frac{\mu_{W}{}^{2}}{2\pi }\int_{0}^{T}Z\left(\phi \left(\tilde{t}\right)\right)\delta \left(\tilde{t}-t\right)d\tilde{t}\\ & =\frac{\mu_{W}{}^{2}}{2\pi }Z\left(\phi \left(t\right)\right) \end{array}$$ (7)

Therefore, by using sequential perturbations with Dirac delta function auto-correlation, the PRC *Z*(*ϕ*) can be obtained from the average of the product between the phase variation Δ_*i*_ and the perturbation *I*_*i*_(*t*).

#### Modified WSTA method using the cyclic nature of the PRC

In the original WSTA method [56], the PRC for one walking cycle is obtained from the superposition of (weighted) perturbations separated by one cycle. In later research, a modified WSTA method that uses perturbations separated by multiple cycles, instead of by just one cycle, was proposed [57]. By using this procedure, the cyclic nature of the phase response curve could be included and the precision of the estimation was reported to be improved. Therefore, in the present research, we use this modified WSTA method to estimate the PRC.

The procedure of the modified WSTA method is as follows: (1) Separate the perturbations by multiple cycles, instead of by just one cycle. (2) Estimate the PRC for multiple cycles from the superposition of weighted perturbations. (3) Determine a proper PRC for one cycle from the PRC of multiple cycles. In order to determine the proper PRC of one cycle, in this research, the candidate multi-cycle PRCs are calculated for separations of 3 to 6 cycles. Then, from the total 18 candidate PRCs of one cycle, the PRC with the smallest variation in all trials of each subject (5 PRCs, as described in the subsection (D)) is selected.

### C. Experiment

In order to consider the characteristics of the rhythm response of human locomotion and the effectiveness of the WSTA method for that purpose, we perform the walking experiment with perturbation and estimate the PRC using both the impulse method and the WSTA method. In the experiment, human subjects walk on a treadmill (ITR3017, BERTEC corporation) with velocity 1.0 m/s, and a perturbation is applied by sharply changing the belt speed of the treadmill. Perturbation is applied under two conditions: (1) sequential impulse perturbation, in which the next disturbance is applied within one walking cycle, and (2) intermittent impulse perturbation, in which the next disturbance is applied after several walking cycles. In each perturbation, the velocity is increased and decreased. The experimental data have been described in another paper [58]. This previous paper analyzed the variation of COM, limb motion, and intersegmental coordination for each walking cycle, while the present paper focuses on the characteristic of walking rhythm.

**Fig 1:**
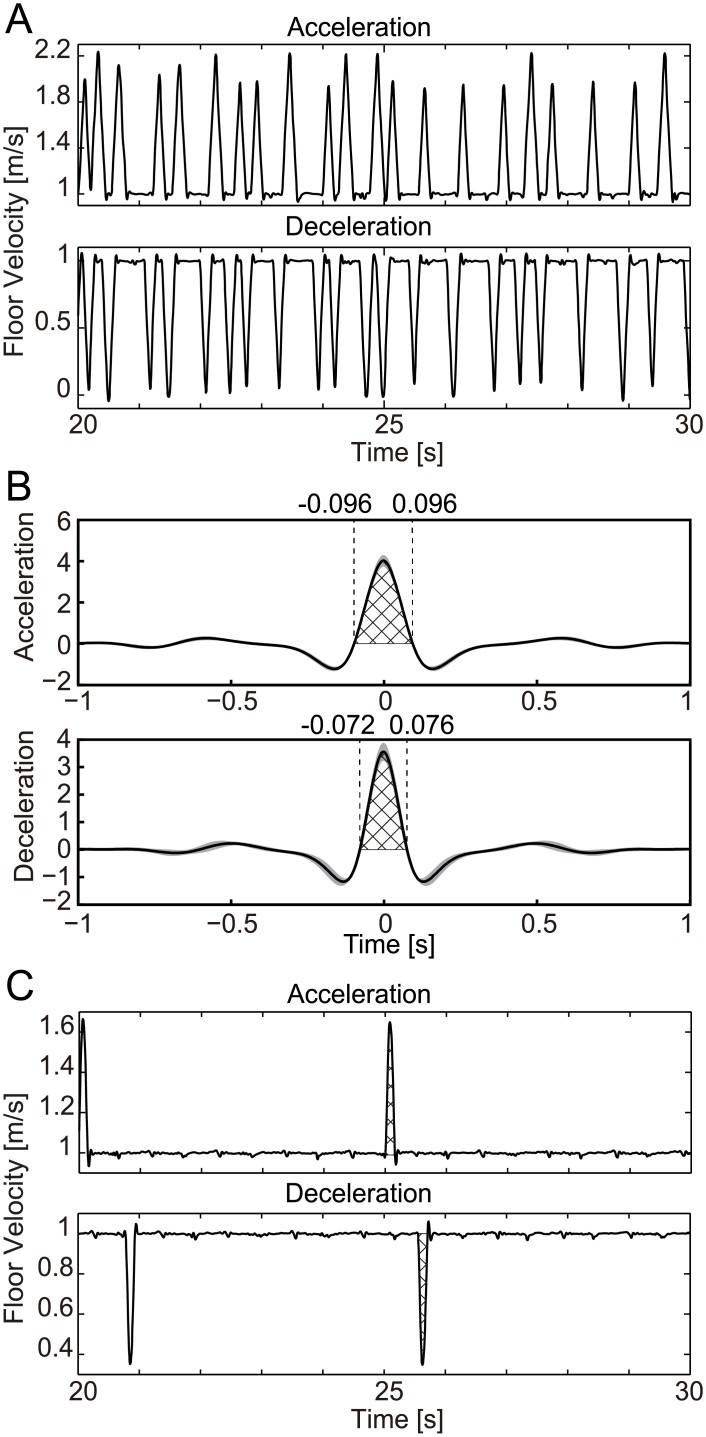
Characteristics of floor perturbation. (A) Time series of treadmill velocity with sequential impulse perturbation used for the WSTA method. Displayed data are for 10 seconds chosen from 180 s of 1 experimental trial of 1 subject. (B) Auto-correlation function of the perturbation for the WSTA method. Displayed data are average (black line) and standard deviation (gray area) of all trials of all subjects. The cross-hatched area is used for the estimation of the size of auto-correlation function. (C) Time series of treadmill velocity with intermittent sequential impulse perturbation used for the Impulse method. Displayed data are for 10 seconds chosen from approximately 60 s of 1 experimental trial of 1 subject. The cross-hatched area is used for the estimation of the magnitude of the impulse perturbation.

In sequential impulse perturbation, the acceleration perturbation increased the belt speed by about 1.2 m/s, and the deceleration perturbation decreased the belt speed by about 1.0 m/s. The belt speed changed linearly over 0.1 s until the desired speed was reached and then returned to the original speed (1.0 m/s) in the following 0.1 s. Fig 1A shows the changing velocity of the floor, and Fig 1B shows the auto-correlation function of the perturbation. Measurement began after the investigator had visually confirmed the attainment of stable walking, and the first perturbation was applied 10 s later. The interval of the perturbation was randomly determined with a maximum interval of 0.5 s and a minimum interval of 0 s. Desired floor velocity is set at the value of perturbation, namely, 2.2 m/s for acceleration and 0 m/s for deceleration after the selected interval. Even if the next perturbation time arrives before the belt reaches the desired speed, meaning that the interval is less than 0.1 s, the desired speed is reset to the same value for perturbation. As a result, the belt provides one perturbation for earlier setting of the belt speed, and behaves as if the latter setting is neglected. Each trial lasted for 180 s, and trials were repeated 15 times. The number of walking cycles for each subject was 2430–2563 cycles for acceleration perturbations and 1925–2261 cycles for deceleration perturbations.

In intermittent impulse perturbation, the acceleration perturbation increased the belt speed by about 0.6 m/s, and the deceleration perturbation decreased the speed by about 0.6 m/s. The belt speed changed linearly over 0.1 s until the desired speed was reached and returned to the original speed (1.0 m/s) in the following 0.1 s. Fig 1C shows the changing velocity of the floor. Each trial lasted approximately 60 s. Measurement began after the investigator had visually confirmed the attainment of stable walking, and perturbations started 10 s later. The perturbation was intermittently applied approximately every 5 s, and after 10 repetitions of the perturbation, the trial ended. A total of 25 trials of acceleration perturbation and 25 of deceleration perturbation were conducted for two subjects, and 15 trials for both perturbations were conducted for all the other subjects. The number of walking cycles was 805–1257 cycles for acceleration perturbations and 780–1248 cycles for deceleration perturbations.

The amplitude of the perturbation was determined in a preliminary experiment so that the maximum amplitude perturbation could be applied without the subjects losing their balance. In the preliminary experiment, the amplitude of the change in velocity was gradually changed, and the subject was asked about his walking comfort following each increment. When the subject felt likely to lose his balance, the increase in the amplitude of perturbation was terminated and that value was selected as the amplitude of the perturbation. As an exception, the deceleration perturbation in condition 1 (sequential impulse perturbation) was determined when the amplitude of the velocity change reached 1.0 m/s. Because the velocity becomes 0 m/s at this time point, any further deceleration would change the direction of movement. The perturbation was applied so that the frequency distribution would be uniform at any phase. The uniformity of the perturbation was confirmed by a Rayleigh test in previous research [58].

All subjects were healthy men (*n* = 11). Five subjects (age = 21–23 years; weight = 53–77 kg; height = 167–177 cm) were tested under condition 1 (sequential impulse perturbation) and eight (age = 21–23 years; weight = 50–80 kg; height = 161–182 cm) under condition 2 (intermittent impulse perturbation). Two of the subjects were tested under both conditions.

A motion capture system (MAC3D Digital RealTime System; NAC Image Technology, Inc.) was used to measure the motion. Reflective markers were attached to the subjects’ skin over several body landmarks on both the left and right sides: head, upper limit of the acromion, greater trochanter, lateral condyle of the knee, lateral malleolus, second metatarsal head, and heel. The sampling frequency was 500 Hz. The belt speed of the treadmill was measured by a rotary encoder. The resolution of the encoder was 3600 p/r, and the pulse of the encoder was measured by a counter with a sampling frequency of 500 Hz.

### D. Analysis

From the time series of motion-capture data, touch-down timing is found as the timing when the heel is at its lowest position, and the walking duration under the perturbed condition is calculated. Then the variation of phase Δ_*i*_ after the perturbation is calculated from the difference between the duration of walking under perturbation and that of steady-state walking.

From the measured velocity of the treadmill belt, floor perturbation *I*_*i*_(*t*) rad/s is calculated in two steps. First, a time series of floor perturbations in m/s is calculated by removing the average belt speed $\bar{v}$ m/s from the measured belt speed under perturbation *v*_*i*_(*t*) m/s. Second, the calculated floor perturbation ($v_{i}\left(t\right)-\bar{v}$ m/s) is converted into the effect on the phase rad/s. Because average stride length *x* m for one cycle (2*π*rad) with average cycle duration *T* s is obtained as $x=\bar{v}T$, conversion from meters per second to radians per second is done by multiplying $2\pi /\left(\bar{v}T\right)$. As a result, the floor perturbation *I*_*i*_(*t*) is $I_{i}\left(t\right)=2\pi \left(v_{i}\left(t\right)-\;\bar{v}\right)/\left(\bar{v}T\right)$ rad/s.

The amplitude of the perturbation *μ*_*W*_ in the sequential impulse perturbation is obtained from the auto-correlation function (Fig 1B). In this research, the auto-correlation function displayed in Fig 1B is approximated by a delta function, and *μ*_*W*_^*2*^ from Eq (6) is set to be the area under the center of this function where its value is positive (cross-hatched area in Fig 1B). The calculated amplitude of the perturbation *μ*_*W*_ was 0.68 (0.03) in acceleration perturbation and −0.58 (0.04) in deceleration perturbation. Here, the values are the averages (standard deviations) of all the measured data.

The amplitude of the perturbation *μ*_*I*_ in the intermittent impulse perturbation is obtained from the magnitude of change in belt speed. The deviation between the (changing) belt speed during perturbation and average speed of unperturbed walking is calculated and its area (i.e., the cross-hatched area of Fig 1C) is used as *μ*_*I*_. The calculated amplitude of the perturbation *μ*_*I*_ was 0.065 (0.01) for acceleration perturbation and -0.066 (0.01) for deceleration perturbation. Here too, the values are the averages (standard deviations) of all the measured data.

The small variations in floor velocity seen approximately every 0.5 s in Fig 1A and 1C are due to changes in belt speed due to touch-down of the right and left legs; touch-down affects the belt tension, temporally changing the belt speed even when the belt is controlled by a servo motor. The amplitude of this variation is approximately 0.02 m/s at maximum. In comparison to the provided perturbation (which exceeds 0.6 m/s), this variation is small. Moreover, it is approximately 2% of the steady speed 1.0 m/s. Therefore, this small variation of speed is ignored in this research.

The PRC estimated by the WSTA method is obtained by multiplying the perturbation *I*_*i*_(*t*) and variation of phase Δ_*i*_ for each cycle and averaging them based on Eq (7). Here, the data points of perturbation *I*_*i*_(*t*) for each cycle is arranged to be of same size (500 points) using linear interpolation for averaging. Moreover, in order to estimate the variation of PRC for each subject, the 15 trials for each subject are divided into 5 groups (3 trials for each group), and 5 PRCs are estimated for each subject. In this research, because 1 trial is walking for 180 s, each PRC is calculated from 540 s of walking motion.

### E. Evaluation of the properties of the human phase response based on the estimated PRC

Using the obtained PRC, the characteristics of the phase resetting of walking are considered. For this purpose, variation of phase *ϕ* against a certain perturbation *I*(*t*) is estimated using Eq (1) and the obtained PRC. Then this characteristic is displayed as a phase transition curve (PTC [28, 59, 60]), which expresses the relation of the phase *ϕ*_*n*_ before the perturbation and the phase *ϕ*_*n*+1_ immediately after the perturbation.

The phase *ϕ*_*n*+1_ after the perturbation has a relation with the amount of phase shift *η*(*ϕ*_*n*_) for the perturbation applied at phase *ϕ*_*n*_:

$$\phi_{n+1}=\phi_{n}+\eta \left(\phi_{n}\right).$$ (8)

The amount of phase shift by a perturbation at *ϕ*_*n*_ can be calculated as the response to perturbation by the Dirac delta function at *ϕ*_*n*_ (*I*(*t*) = *μδ*(*t*−*s*)), where *s* represents the time corresponding to phase *ϕ*_*n*_. From Eq (1), the phase equation becomes

dϕ=(ω+Z(ϕ)μδ(t−s))dt. (9)

By integrating both sides over the duration of the perturbation, we obtain

$$\phi_{n+1}-\phi_{n}=0+Z\left(\phi_{n}\right)\mu ,$$ (10)

and then the variation of phase can be calculated as

$$\eta \left(\phi_{n}\right)=\mu Z\left(\phi_{n}\right).$$ (11)

Here, *μ* is the magnitude of the perturbation applied as a Dirac delta function, and *μ*_*I*_ used for the impulse method is the variable with the same meaning. For the intermittent impulse perturbation used in the impulse method, the magnitude of the perturbation is set at the maximum value for which stable walking can be maintained, thus a value larger than *μ*_*I*_ will induce a behavior different from stable walking. In particular, the perturbation used in the WSTA method is different from the Dirac delta function, and its magnitude *μ*_*W*_ is larger than *μ*_*I*_. Therefore, using the value of *μ*_*W*_ as *μ* would not be proper. In this research, thus, the values of *μ*_*I*_ appearing in the last subsection (i.e., 0.065) for an acceleration perturbation and −0.066 for a deceleration perturbation, are used for the values for *μ*.

Moreover, this transition characteristic is calculated for 500 phases uniformly distributed before the perturbation, and the distribution of these phases after the perturbation is displayed in a histogram. Based on this histogram, characteristic phases for phase resetting are derived.

## Results

### PRCs identified using the WSTA and impulse methods

**Fig 2:**
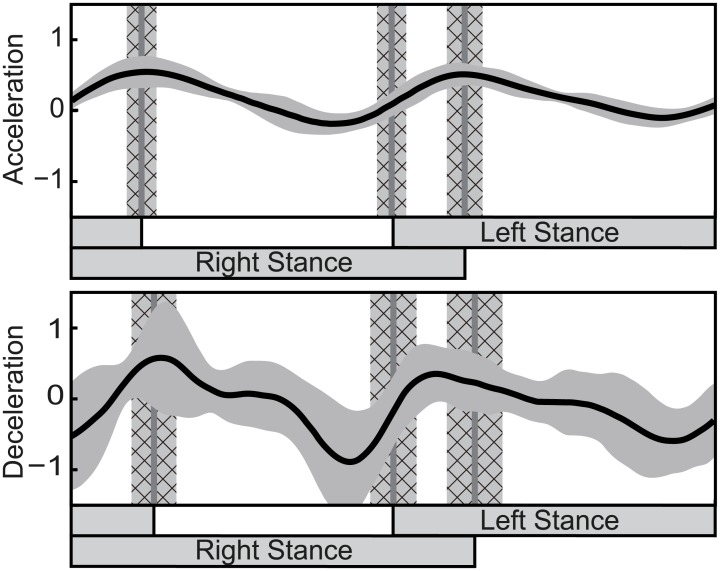
Estimated phase response curve (PRC) using the WSTA method. The black curve is the average PRC for all the trials of all the subjects and gray area around the average represents the standard deviation. The gray lines and gray cross-hatched areas are the touch-down/lift-off timings and their standard deviations.

Fig 2 shows the human PRC identified using the WSTA method. The horizontal axis represents the phase of one cycle starting from the heel-contact of the right leg, and the black curved line represents the PRC. The three gray vertical lines in the figure represent the time of left leg lift-off, left leg touch-down and right leg lift-off, and the cross-hatched gray areas represent their standard deviations. The figure shows that the PRC has a negative peak at touch-down and a positive peak just before lift-off. Patterns obtained from acceleration and deceleration perturbations have a similar shape: values at the peak timings are negative around touch-down and positive around lift-off timing. Here, the relation between the positive and negative values and the property of phase reset (phase advance or delay) depends on the perturbation *I*(*t*), as shown in Eq (1). In general, positive *I*(*t*) by acceleration perturbation and positive PRC generates phase advance, and negative *I*(*t*) with deceleration perturbation with positive PRC generates phase delay. Such variation of the phase can be discussed using PTC for more detail. One important characteristic of the obtained PRC is that the PRC during single support phase of deceleration perturbation is similar to the PRC previously obtained by directly pulling the swing leg [26, 27]. This result supports the validity of the obtained PRC.

**Fig 3:**
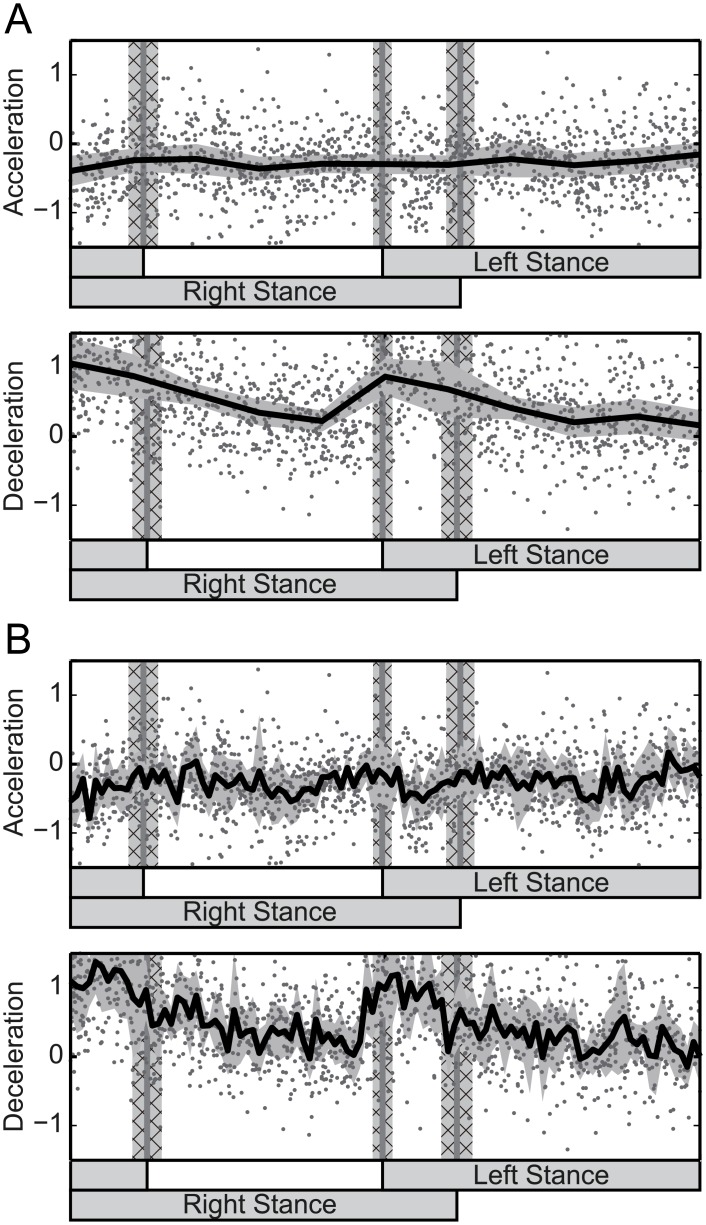
Estimated phase response curve (PRC) using the impulse method. The gray points are the phase variation for each impulse perturbation. Data from all trials of all subjects are displayed. The black curve is an average of the points within (A) 10% and (B) 1% of the walking cycle. The gray area around the curve is the standard deviation. The gray lines and gray cross-hatched areas are touch-down/lift-off timings and their standard deviations.

Fig 3 shows the human PRC identified using the impulse method. Each gray point in the figures is the amount of change from one impulsive perturbation at that phase, and averaged distributions of the points in the range of 1/10 of a walking cycle (Fig 3A) and 1/100 of a walking cycle (Fig 3B) are shown as curved lines in the figures. The patterns of the deceleration perturbation contain, as in the PRC of the WSTA method, a positive peak during the double-support phase and a negative peak before the touch-down phase. On the other hand, patterns of the acceleration perturbation do not show clear patterns: the range between the positive and negative peaks of the pattern is similar or smaller than the standard deviation. Moreover, by comparing Fig 3A and 3B, we see that the increased temporal resolution in Fig 3B increases the temporal deviation of the pattern, and the pattern become unstable.

Overall, in order to investigate the phase-dependent (temporal) characteristics of the PRC, the PRC obtained by the WSTA method has the advantage of a low temporal deviation. Therefore, the following analysis to investigate the characteristics of the human walking rhythm is based on the PRC obtained by the WSTA method.

### Characteristics of the PRC obtained by the WSTA method

The PRC obtained by the WSTA method (Fig 2) has a similar shape for the acceleration and deceleration perturbations: both curves have a positive peak around the lift-off phase and a negative peak before the touch-down phase. The characteristic of the curve is given by the relation between the peak timings of the curve and the timings of the touch-down and lift-off phases. This is then used, in particular, to investigate the effect of the different perturbations (acceleration and deceleration) on the PRC.

**Fig 4:**
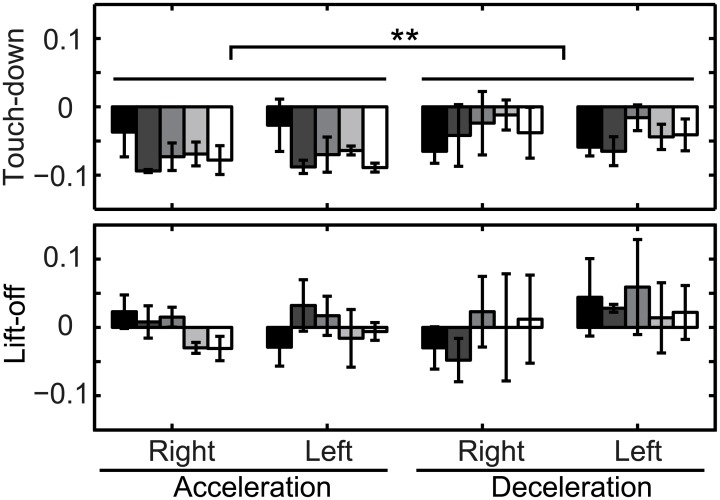
The deviation (seconds) between the peak timing of phase response curve and the nearest touch-down or lift-off timings. The different color bars show the results for each subject. Each bar represents the average and standard deviation of 5 PRCs obtained for each subject.

In order to evaluate the peak timings in relation to the touch-down and lift-off phases, the peak timing is sought in a range of 10% of a cycle before and after the touch-down and lift-off phases. Fig 4 shows the resulting peak locations of each subject, and Table 1 shows the average and standard deviation of all the trials of all subjects. The negative peak around the touch-down phase is located, in the acceleration perturbation, at approximately 70 ms before the touch-down phase and, in the deceleration perturbation, approximately 40 to 50 ms before the touch-down phase. In contrast, positive peaks were located closer to their reference timing, specifically, within −10 to 30 ms of the lift-off phase in both acceleration and deceleration perturbations. In order to investigate the difference in the effects of the acceleration and deceleration perturbations on the peak timing, a 2-way ANOVA of direction and subject was performed. The negative peak timings depend significantly on the direction (p < 0.01), but the positive peak timings are not significantly different (p = 0.11).

**Table 1:** Average (standard deviation) of peak phase around touch-down and lift-off phase. Values are in seconds.

	Acceleration		Deceleration	
	right	left	right	left
Touch-down	-0.07 (0.03)	-0.07 (0.03)	-0.04 (0.04)	-0.05 (0.03)
Lift-off	0.00 (0.03)	0.00 (0.04)	-0.01 (0.06)	0.03 (0.05)

The overall result is that the PRC obtained by the WSTA method is found to show a negative peak (approximately 40 ms) before the touch-down phase, and a positive peak around the lift-off phase. The shapes of the PRCs are similar for both directions of perturbation, but the location of the negative peak before the touch-down phase depends significantly on the direction.

### Phase transition of walking estimated by the obtained PRC

**Fig 5:**
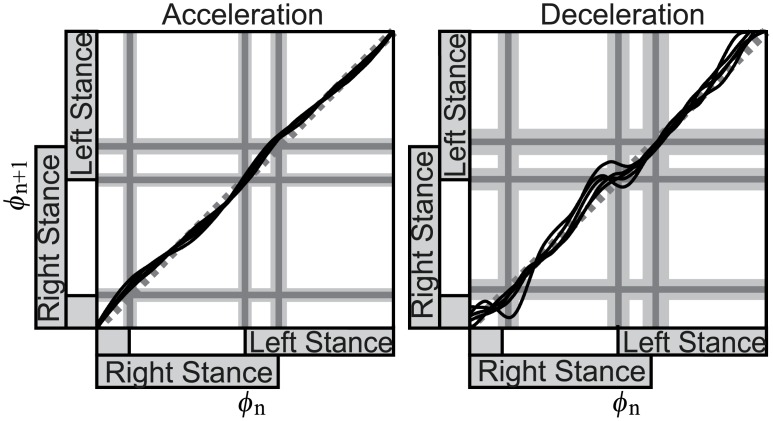
Phase transition curve. The black curves are the average phase transition curves of each subject. The gray dotted line is the line of *ϕ*_*n*+1_ = *ϕ*_*n*_. The gray lines and gray areas are the timings of touch-down/lift-off and their standard deviations.

In order to consider the variation of walking rhythm caused by the perturbations, the PTC that represents the relation between the phases before and after the perturbation [29] is drawn using the PRC obtained by the WSTA method (Fig 2). Fig 5 shows the calculated PTC. Here, the amplitude of perturbation used for the calculation of PTC is *μ*_*I*_, as described in the Methods section (E). In the figure, the result of the acceleration perturbation was sinusoidal, with the touch-down and mid-single support phases as nodes. The result of the deceleration perturbation was step-like with the angle changing sharply around the touch-down phase. In the PTC, lines with an incline higher than that of the line *ϕ*_*n*+1_ = *ϕ*_*n*_, that is, those where *ϕ*_*n*+1_ > *ϕ*_*n*_, indicate an acceleration of the rhythm (phase advance) and those with a lower incline (i.e., *ϕ*_*n*+1_ < *ϕ*_*n*_) indicate a deceleration of the rhythm (phase delay). From the figure, it can be seen that the lowest inclines are found in mid-stance phase for the acceleration perturbation and at touch-down for the deceleration perturbation. Therefore, the walking rhythm is modified at these phases.

**Fig 6:**
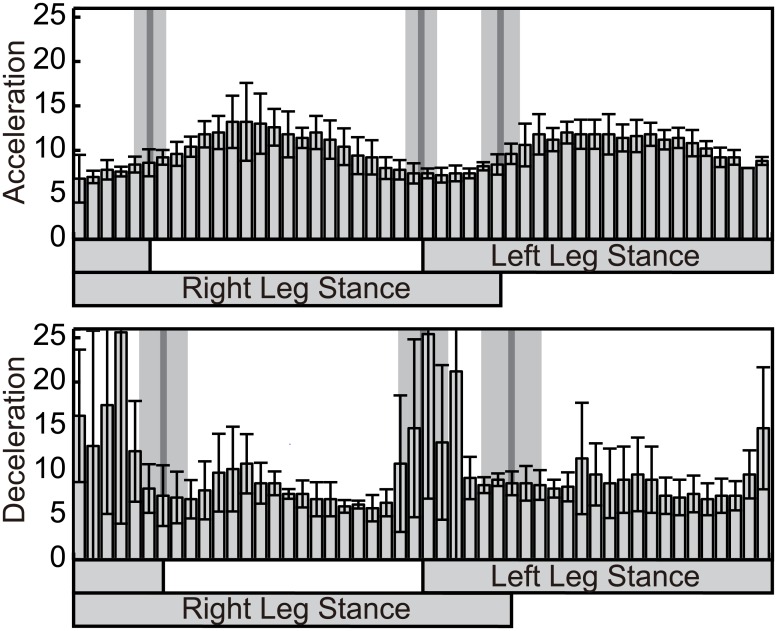
Histogram of the walking phase after transition by the perturbation. The distribution of post-transition phases for 500 points uniformly selected before the transition are displayed using a histogram with 50 bars. The results are shown using the average and standard deviation of all subjects. The gray lines and gray areas are timings of touch-down/lift-off and their standard deviations.

In order to investigate the change of walking phase throughout the cycle, 500 pre-perturbation walking phases were uniformly chosen over the walking cycle, and a histogram of the location of these phases after the transition is shown as Fig 6. From the figure, it can be seen that phases after the perturbation are concentrated on the mid-single support phase for the acceleration perturbation, and around the touch-down phase for the deceleration perturbation. Therefore, human motion is found to slow down around the mid-single support phase for the acceleration perturbation and around the touch-down phase for the deceleration perturbation.

**Fig 7:**
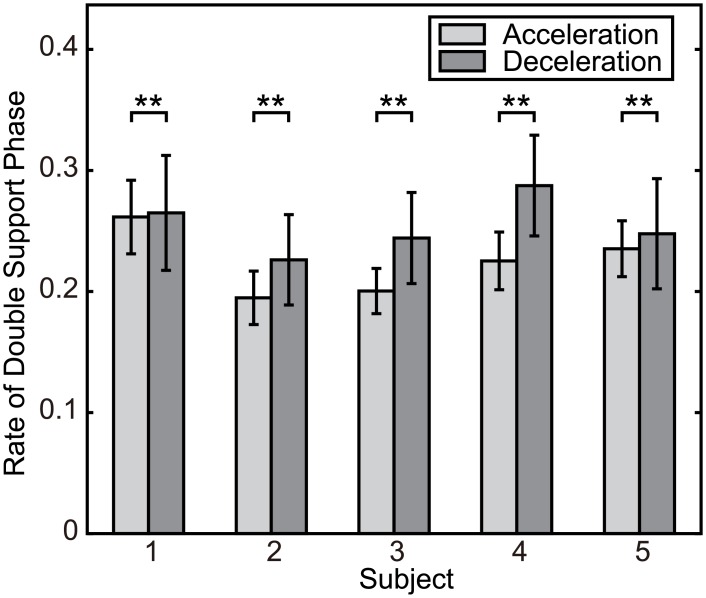
The ratio of the length of the double support phase to the length of the whole walking cycle. The bars show the average and standard deviation for each subject.

The existence of a different response to the acceleration and deceleration perturbations is confirmed by the ratio of the duration of the double support phase to that of the whole walking cycle. If the single support phase is extended for the acceleration perturbation and the phase is modified during the double support phase for the deceleration perturbation, the relative length of the double support phase for the deceleration perturbation will be longer than that for the acceleration perturbation. Fig 7 shows the ratio of the length of the double support phase to that of the whole walking cycle for acceleration and deceleration perturbations. In order to compare the ratios, the significance of the difference was calculated using a *t*-test, and the double support phase ratio was confirmed to be larger for deceleration perturbation (*p* < 0.01) for all subjects. The same result was also obtained by using Welch’s *t*, which does not assume uniformity in variance, and a 2-way ANOVA of perturbation direction and subject showed a significant difference depending on the direction of the perturbation. From these results, a different modification of motion is confirmed to occur depending on the perturbation, and this modification of motion is shown in Fig 6.

## Discussion

In order to investigate the human control strategy of changing walking rhythm in response to disturbance, we applied perturbations over the entire walking cycle by changing floor speed, and estimated the PRC. For estimating the PRC from responses to weak perturbation, in particular by indirect effects on the swing leg, we employed a WSTA method that uses sequential impulse perturbation for the estimation of human rhythm for the first time. Experiments were performed by applying sequential impulse perturbations to humans walking on treadmill, and the PRC was calculated from the floor velocity and human motion.

The results obtained by this research are as follows: (1) The PRC estimated by the WSTA method with floor perturbation has clear peaks for both acceleration and deceleration perturbations, and the shape was smooth and periodicity was guaranteed. In contrast, the PRC estimated by the impulse method with floor perturbation showed similar peak timing for deceleration perturbations, but the peak timing for acceleration perturbations was not clear. Moreover, at high temporal resolution the shape became unstable in the impulse method. Thus, the WSTA method has an advantage over the impulse method, particularly for investigating the temporal characteristics of the PRC. (2) The PRC estimated by the WSTA method has a negative peak before the touch-down phase and a positive peak around the lift-off phase for both acceleration and deceleration perturbations. Moreover, the estimated PRC by deceleration perturbation has a similar shape with previous estimates [26, 27] using direct disturbance to the swing leg. (3) By estimating the phase transition due to perturbation using the PRC obtained by the WSTA method, the phase after the perturbation was found to be concentrated around the mid-single support phase for acceleration perturbations, and around the touch-down phase for deceleration perturbations.

In the following discussion, we first discuss the validity of the experimental condition and the results, and then discuss the relevance of the obtained results to previous studies on the physiological mechanisms of response to disturbances. Through these discussions, we consider the relevance of these results to human walking control.

### Experiment and results 1: Amplitude of perturbation

To investigate the phase response for the entire walking cycle, this research provided perturbation by changing the velocity of a treadmill. This provided perturbation for both the swing and stance legs, but at the same time the amplitude of the perturbation decreased from that in previous studies on walking PRC [26, 27]. As a result, the PRC obtained by the impulse method was insufficiently precise and the WSTA method was required. Here, we consider whether we really cannot use perturbation with higher amplitude that enables the impulse method for obtaining precise PRC, and we discuss the validity of the amplitude of perturbation used in the present research.

**Fig 8:**
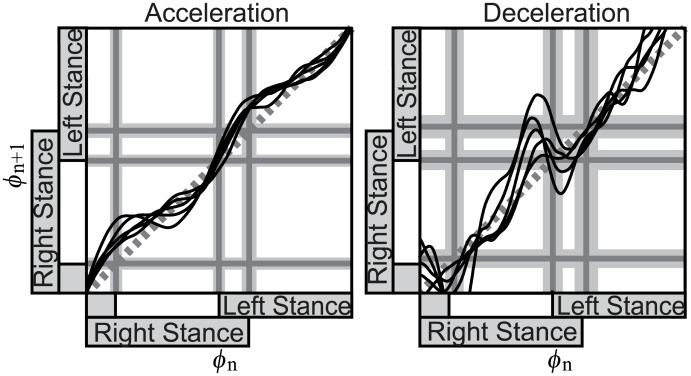
Phase transition curve with a stronger perturbation than in the actual experiment (3*μ*_*I*_). The black curves are the average phase transition curves of each subject. The gray dotted line is *ϕ*_*n*+1_ = *ϕ*_*n*_. The gray lines and gray areas are timings of the touch-down and lift-off and their standard deviations.

**Fig 9:**
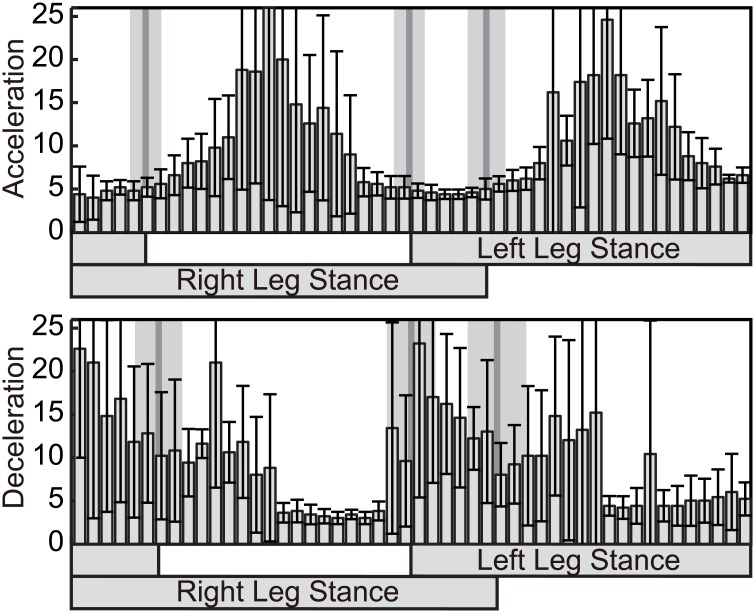
Histogram of the walking phase after transition by the perturbation. Perturbation is stronger than in actual experiments (3*μ*_*I*_). The results are shown using the average and standard deviation of all subjects. The gray lines and gray areas are timings of the touch-down and lift-off and their standard deviations.

We first review how to determine the amplitude of perturbation. This was done in a preliminary experiment, in which the amplitude of velocity change was gradually increased while inquiring the subject not to exceed limits for maintaining balance (see the Methods section (C) for details). We then considered what will happen if a stronger perturbation is provided. This can be estimated from the PTC. Figs 8 and 9 are respectively the PTC and the histogram of the phase after the transition, with three times larger amplitude (3*μ*_*I*_) of the perturbation than used in actual experiments for the impulse method. By focusing on the results of deceleration perturbation in Fig 8, negative slopes can be found, while the actual experimental results (Fig 5) were stepwise. The negative slope means that the walking rhythm reverses, and the result of the phase transition (Fig 9) was rather complex. It is doubtful whether humans can achieve such a complex phase transition, implying that an experiment with such a strong perturbation would be difficult.

The amplitude of sequential impulse perturbation used for the WSTA method was higher than that of intermittent impulse perturbation used for the impulse method in the present experiment. The effect of muscle tonus can be considered as a possible reason for this phenomena. When the perturbation was provided sequentially, muscle tonus increased in response to the previous disturbance, while it had already recovered when the perturbation was applied intermittently. Higher muscle tonus thus reduced the effect of the perturbation, and a higher amplitude of perturbation can be provided in sequential perturbation.

Finally, we consider whether sufficient amplitude of perturbation was applied in the experiment. This is justified by the shape of the PRC obtained by the WSTA method. PRC for deceleration perturbation during the single-support phase (the only comparable phase) was similar to that observed in previous studies [26, 27]. This indicates the perturbation was sufficiently strong for estimating with the WSTA method, and the amplitude of the perturbation was therefore considered to be appropriate.

### Experiment and results 2: Differences in standard deviation depending on the perturbation

The results of the experiment and analysis indicate the existence of phase resetting in response to perturbations during walking. For deceleration perturbation, rhythm resets around the touch-down timing. For acceleration perturbation, this occurs around the mid-single support phase. As a characteristic of the obtained PRC (Fig 2), we also notice that the size of standard deviation (SD) varies between acceleration and deceleration perturbations. Considering what caused this difference in SD, we first considered the tendency PRC differences through trials. This suggested that the PRC of deceleration perturbation differed more in the time direction than did that of acceleration perturbation, despite similar patterns. This property is reflected in Fig 2, as its SDs of touch-down and lift-off are large. This variation in time is considered to occur due to variation in the touch-down timing. Because the calculation of SD and drawing of the figure is performed with the touch-down as the initial timing, the variation of touch-down timing thus affects the variation of all timings. Another question is why the variation of touch-down timing in deceleration perturbation is larger than that of acceleration perturbation. This can be estimated from Fig 6. In deceleration perturbation, the phase is shifted around the touch-down timing, and so changed depending on the applied perturbation. In contrast, the mid-support phase is the mainly shifted for acceleration perturbation. This difference in phase resetting is considered to generate different SD sizes between acceleration and deceleration perturbations.

### Physiological mechanisms 1: Comparison of the results with physiological mechanisms

By comparing the obtained results, phase resetting around the touch-down and mid-single support phases, with a phase control mechanism previously reported in nervous systems through mechanical and electrical stimulation, we discuss the relevance of the obtained results with the physiological mechanisms.

By electrical stimulation to the swing legs (stumbling corrective reaction) in cats [1, 61] and humans [62, 63] and mechanical stimulation in humans [3, 4], response to the stimulation is reported to reverse depending on the stimulation timing (reflex reversal) [61]. Namely, stimulation at early swing phase enhances flexor muscles and extends swing phase (elevating strategy), while stimulation at late swing phase enhances extensor muscles and advances the touch-down timing (lowering strategy) [2]. Fig 6 shows that the mid-single support phase extending in response to acceleration perturbation and the touch-down timing advancing in response to deceleration perturbation, which are considered to respectively correspond to the elevating strategy and lowering strategy. By comparing the joint response to the cutaneous reflex and elevating strategy, the stumbling corrective reaction is reported to be originated by cutaneous reflex [7, 8]. (Note that some studies also reported the contribution of proprioceptive afferent [64, 65].) Electrically stimulating the cutaneous superficial peroneal nerve of a decerebrate cat during fictive locomotion is shown to enhance extensor activity of the hip and knee and flexor activity of the hip [66], and intracellular analysis showed that the motoneuron is di- and tri-synaptically excited [11]. From these observations, the response originating from the cutaneous afferent during swing phase is considered to affect the rhythm of CPG [11, 12].

As a response to the proprioceptive afferent, Conway et al. [5] stimulated the group I afferent of the knee and ankle during the flexion phase, and they found that flexion activity terminated and transited to the extension phase, while the extension phase is increased by the stimulation. This response is reported to be generated at the premotor level, and this is also considered to affect the rhythm generator [5, 9]. In this way, both cutaneous and proprioceptive afferents affect the CPG, and shift the walking rhythms.

We next discuss mechanisms through which these afferents and rhythm control systems generated the obtained phase resetting at the mid-single support phase and around the touch-down phase. We first consider the phase reset around touch-down. Considering the timing of phase reset, a lowering strategy that advances the touch-down from swing phase and a proprioceptive response that affects the transition between touch-down and lift-off [6, 67] are considered to be involved. Here, we also focus on the process of phase transition. Fig 5 shows that acceleration and deceleration perturbations affected not only in the timing of the peak but also the shape of the response; the response to a deceleration perturbation is stepwise and almost horizontal at the touch-down phase, while the response to an acceleration perturbation centered at the mid-single support phase is a smooth, sinusoidal shape. The characteristic of a horizontal transition curve indicates that the phase after the perturbation changed drastically depending on the touch-down event. A possible reason for this drastic change of phase is a rule-based phase reset [41]. This also corresponds to the behavior of dynamical simulations with phase resetting at touch-down timing [42, 43, 46]. From these observations, phase reset around touch-down is considered to involve the phase advance due to the cutaneous afferent and proprioceptive originated drastic phase reset at touch-down.

We next consider the mid-single support phase in response to the acceleration perturbation. The first question when considering this phenomenon is whether it is a response to the swing or the stance leg. Here, we recall that the obtained PRC during the single-support phase has a similar shape with that obtained by pulling the swing leg [26, 27]. This result supports the idea that the perturbation affected the swing leg and the stumbling corrective reaction worked. In swing phase, response to the proprioceptive afferent is lowered by presynaptic inhibition from the contralateral leg [15, 16]. This presynaptic inhibition is reported to affect the group II and group Ib afferents, but not the group Ia and cutaneous afferents [14]. In fact, stumbling corrective reaction is generated by small tactile sensations such as air puffs [1]. Therefore, an elevating strategy originating from a cutaneous afferent is involved in the response to the acceleration perturbation.

Finally, we discuss why such a difference occurred between acceleration and deceleration perturbations. Because perturbation is provided from the floor in this research, we focus on difference in motion around the stance leg. In deceleration perturbation, the stance leg is relatively pulled forward, and the body rotates backward. The force for maintaining posture is centered at the heel. This reaction is considered to be the reason why phase resets around the heel contact mainly occurred for deceleration perturbation. In contrast, acceleration perturbation rotates the body forward, and thus the phase reset around the heel is not considered to have occurred.

### Physiological mechanisms 2: Relation with permanent rhythm shift

Rhythm control of humans and animals includes transient rhythm shifts for disturbances and permanent rhythm shifts for walking with different left and right speeds, such as in curved walking [68, 69]. Here, relevance of the observed behavior in the present experiment with this permanent rhythm shift is discussed. Permanent rhythm shifts have been studied through walking on split-belt treadmills with right and left belts moving at different speeds [70–72]. By changing the speed of one belt, intra-limb characteristics of movement changes soon after the changing belt speed (early adaptation), and inter-limb coordination gradually changes after approximately 1 min (late adaptation) [70]. To consider the relation between the phase reset and these early or late adaptations, Fujiki et al. performed two experiments using bipedal robots [51, 52]. In these experiments, robots are activated mainly with feedforward control, and phase reset at a touch-down phase similar to that observed in Fig 6 is used (see also [46] for detailed control procedures). As a result, stable walking is realized by phase reset and early adaptation is observed, but late adaptation cannot be realized [51]. Next, an error learning model of touch-down timing is added based on the error learning algorithm in the cerebellum [73, 74], and shows that late adaptation including after-effects similar to human behavior is realized [52]. From these results, the mechanism for permanent rhythm shifts for walking includes transient rhythm control for CPG and learning mechanisms in a higher system that transfers the generated transient rhythm shift to a permanent one. Observed phase resets in the preset paper are considered to be related to the lower system around the transient rhythm control of CPG.

### Physiological mechanism 3: Does the experiment of the present research relate to the discussion of flexor dominance?

In previous studies of rhythm control mechanisms, flexor dominance, in which the duration of stance phase changes while the duration of swing phase is fixed, was indicated [75–77]. This characteristic varied depending on the experimental conditions: some concluded that flexor dominance was not essential [12, 78, 79], and others reported that it occurred for spontaneous walking [80, 81]. In the present research, phase resetting at touch-down was found for deceleration perturbations and phase resetting in the single-support phase was also found. Thus it is interesting to consider whether these different phase resets affect flexor dominance.

**Fig 10:**
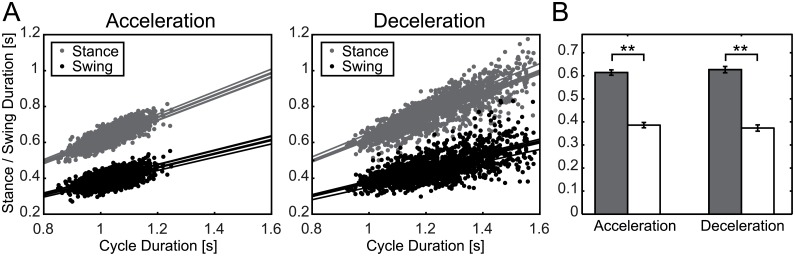
The relationship between cycle length and stance/swing phase. (A) Results of acceleration and deceleration perturbation are displayed as points. Linear regression results for each subject are displayed as lines. (B) Regression coefficient of linear regression. Results are the average and standard deviation for each subject.

To investigate this issue, the relations between swing duration or stance duration and walking cycle are constructed for acceleration and deceleration perturbations, as shown in Fig 10A. Linear regression analysis was performed for each relation and the regression coefficient is shown in Fig 10B. From these figures, it can be seen that both swing duration and stance duration changed depending on the change in walking cycle (every value in Fig 10B is positive). Moreover, because the regression coefficient of stance duration was significantly higher (p < 0.01; *t*-test), flexor dominance that changed the stance duration relative to the swing duration was found to exist. Because these results were found both for acceleration and deceleration perturbations, the characteristic of phase resetting found in the present research is considered to be independent of the characteristic of flexor dominance.

### Prospective future work

The PRC discussed in the present paper contains the phase characteristics for an entire cycle, and through these characteristics the human strategy of rhythm control for any perturbation timing can be investigated. However, this research used only one magnitude for each condition of perturbation, and thus we cannot address issues such as whether a different magnitude of perturbation might induce a different control strategy. To elucidate such an effect, PRCs need to be calculated for various magnitudes of perturbation, including mainly weaker perturbations. However, the S/N rate, that is, the ratio of the changes of cycles due to perturbations over the deviations of walking cycles (which exist even in stable walking) will decrease for weaker perturbations. The results of the present paper show that the WSTA method can provide a more stable phase response curve than can the conventional impulse method. Therefore, the WSTA method is expected to be effective in studies of weaker perturbations. In this way, the approach used the present paper will be a useful method for investigating the rhythm control of humans in a wide range of circumstances.
